# Analysing the behavioural, psychological, and demographic determinants of financial decision making of household investors

**DOI:** 10.1016/j.heliyon.2023.e13085

**Published:** 2023-01-21

**Authors:** Parul Kumar, Md Aminul Islam, Rekha Pillai, Taimur Sharif

**Affiliations:** a School of Banking, Financial Services, and Insurance, Delhi Skill and Entrepreneurship University, India; b Faculty of Applied & Human Sciences, Universiti Malaysia Perlis, Arau, Malaysia; c College of Business Administration, Ajman University, United Arab Emirates; d School of Business, Newman University Birmingham, UK

**Keywords:** Financial literacy, Financial autonomy, Financial attitude, Financial capability, Behavioural finance, Impulsivity

## Abstract

Adding to the behavioural science domain, the principal idea behind the study is to investigate the impact of an array of behavioural, psychological, and demographic factors on financial decision making. The study utilizes a structured questionnaire to collect the opinions of 634 investors using a blend of random and snowball sampling techniques. The partial least squares structural equation modelling has been used to test hypotheses. PLS Predict has been applied to estimate the out-of-sample predictive power of the proposed model. Finally, the multi-group analysis has been applied to assess the differences across gender. Our findings attest the relevance of digital financial literacy, financial capability, financial autonomy, and impulsivity on financial decision making. Additionally, financial capability partially mediates the nexus between digital financial literacy and financial decision making. Also, Impulsivity negatively moderates the relationship between financial capability and financial decision making. The overall results of this comprehensive and unique study portray the influence that various psychological, behavioural, and demographic factors have on financial decision making, favouring the design of a feasible and lucrative financial portfolio to ensure financial well-being of households in the long run.

## 1 Introduction

The rational information processing models in finance show an inclination towards an intuition system where the well informed and financially literate investors make rational and quality financial decision making (FDM) [1] whilst enhancing their economic security and well-being [2,3]. FDM is based on the premise that individuals weigh costs and benefits of a decision (also known as reflective process), which has financial implications and components associated with it. The consensus emanating from behavioural decision sciences is that robust FDM mitigates decision making bias and enables successful attainment of expected decision outcomes [4]. Nonetheless, many individuals are still seen to be ignorant about the extent of their self-unconscious bias towards decision making abilities that differ among individuals and vacillate throughout one's life span [5]. Research points out the complexities associated with evaluating myriad financial products and the conflicting situations investors face in dealing with financial consequences that makes FDM a tough puzzle to solve, altogether [6,7].

Given this background, the cumulative effect of behavioural, psychological, situational, and demographic factors has emerged as promising precedents to FDM [8] while technology-led proliferations in both online resources and innovative investment horizons have facilitated conscious FDM [9]. Early research [10] associated the decision-making quality to two cognitive factors namely deliberation and intuition. Lusardi [11] suggests that sound financial literacy and numerical skills are assumed to be of paramount importance in FDM. Likewise, Luo et al. [12] advocate that the cumulative effects of skills and attitude build financial capability (FC) which in turn impacts FDM. Arifin et al. [13] emphasise financial attitude (FA) as a supporting factor to FDM.

Adding on to the skills mentioned, digital financial literacy (DFL) is assumed to have rationalized financial decisions [14] with personal/behavioural characteristics such as impulsivity and materialistic attitude impacting FDM as well [15]. Nevertheless, Vosylis and Klimstra [16] perceive financial autonomy, a feeling of self-empowerment and confidence in taking independent financial decisions as a vital FDM determinant whereas Gonzalez-Igual et al. [17] emphasise the relevance of education, age, and gender in influencing FDM. Additionally, prudent financial behaviour in the form of consistent expense evaluations, maintenance of contingency reserves [18], budget preparations, low impulsivity, and cost controls [19] also lead to judicious FDM. All these developments in behavioural finance reiterates the importance of considering an integrative approach for better FDM where each system can derive optimal results under varying situations, as suggested by Hochman et al. [20]. More recent additions in the list of FDM drivers are the present economic situation that influences individual financial perception, unconscious bias, and their financial experiences [21].

In light of the above backdrop, we investigate the impact of an array of behavioural factors such as digital financial literacy (DFL), financial capability (FC), financial autonomy (FAUT); cognitive psychological factors namely impulsivity (IMP) and financial attitude (FA) as well as gender on FDM in the Indian economic setting. We investigate the research topic from a developing country perspective and consider India for the empirical investigation. This is because, India was one of the few countries which observed the longest and most stringent lockdowns in the world, with the GDP slumping by 24% causing major upheavals in unemployment rates (up by 24%) and massive slides in household income (down by 46%). Moreover, worsening macroeconomic indicators such as job losses, GDP deteriorations and accelerated inflations, have put working individuals in a financial dilemma, rendering more than 136 million workers financially vulnerable [22] during the pandemic.

From the perspective of extant literature, we offer an extension to the behavioural science domain. This is because, through generations, multidisciplinary schools of thought [23,24] have explored individual decision-making behaviour using both normative and prescriptive approach to decision analysis. This is to comprehend the underlying process and decisive factors in rationalising decisions. While the former attribute robust decisions made by diligent and rationale individuals, the latter considers inherent human limitations and behaviour to unconsciously influence the so-called rationale individuals. Nonetheless, prior studies till date have only considered FDM factors in independence [11,25,26] without investigating their comprehensive effect on FDM, thus leaving a major gap in the FDM research domain. This enhances the novelty of this study by being the first to decipher a new empirical model to comprehend the FDM process.

The current void in literature and invalidity in prior findings serve as the main motivators for conducting a first-hand empirical study on personal, behavioural, and psychological factors affecting financial decision making. Also, given that financial capabilities and cognitive skills fluctuate with gender, further strengthening or weakening the impact of various determinants on FDM, this study investigates the influence of gender related differences on individual FDM.

Altogether, we challenge the rational decision-making behaviour and propose the influence of positive and disempowering factors to achieve four-fold objectives in this study, i.e., firstly, to identify the personal, behavioural, and psychological factors affecting FDM. Secondly, to empirically investigate the association between various behavioural and psychological factors on FDM, aiming at extending the existing literature on FDM. Thirdly, to evaluate the moderating effects of gender and age on the determinants of FDM. Finally, to invoke implications from the results derived and offer recommendations for enhancing individual FDM. To accomplish these objectives, the study utilizes a structured questionnaire to survey the opinions of 634 household investors across the National Capital Territory (NCT) of Delhi in India to decipher the driving factors in FDM.

A comprehensive literature review is done to identify the FDM factors and formulate eight testable hypotheses and the SmartPLS 3.3 is used for testing the hypotheses. The partial least squares (PLS) predict is applied to estimate the sample predictive power. The differences across gender are assessed using the multi-group analysis (MGA) technique. Findings reveal a positive association of DFL, FAUT, and IMP with FDM. Further, we observed a significant positive mediating role of FC in the DFL-FDM nexus.

The remaining paper is structured as follows. Section 2 emphasizes on theoretical background, prior literature, and hypotheses formulation. Section 3 elucidates data and methodology, followed by discussion of results in Section 4. Section 5 concludes the paper with implications and outlines future research prospects.

## 2 Theoretical background, literature review, and hypotheses formulation

FDM has harnessed significant attention in the past decade due to the incongruities evident in financial planning and diminished savings [27]. It is based on the premise that currently individuals have the privilege to choose from a varied set of financial alternatives that ensures wealth maximization. The concept can be rooted to the prospect theory [28], which advocates that an individual performs a cost-benefit analysis (CBA) when exposed to options and chooses those that entail a fair degree of attainment certainty and minimum negative financial implications attached to it.

**Fig. 1:**
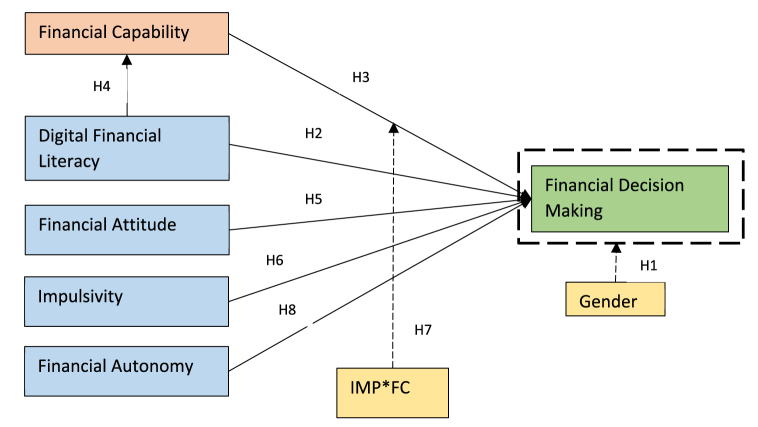
Conceptual model.

That said, the unprecedented changes, complexities, and multidimensional facets of FDM compel us to complement the prospect theory through an earlier model, called decision making theory [29]. The theory emphasizes the futility of predetermined analytics to assist decisions due to conflicting goals and contexts associated with such decisions. This is because, FDM is invariably a cognitive process, hence shows varied patterns based on personality traits (reward seeking or risk averse), velocity and liberty one holds in taking such decisions. Therefore, this paper assumes that FDM as an outcome of behavioural factors such as digital financial literacy (DFL), financial capability (FC), financial autonomy (FAUT); cognitive psychological factors namely impulsivity (IMP) and financial attitude (FA) as well as a demographic factor such as gender in the context of India. The hypotheses and theory to be tested are presented in Fig. 1.

**Fig. 2:**
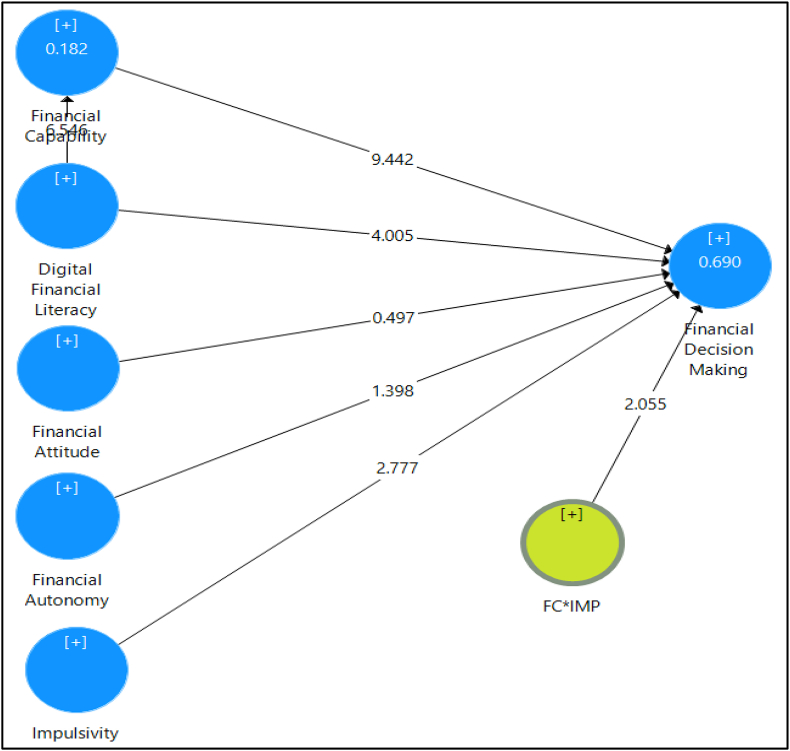
Structural model. *Source: Authors' own estimates*.

### 2.1 Gender

Extant research has deeply delved into the gender influence on FDM [[30], [31], [32], [33]]. The consensus is that women are risk averse [17]. They exhibit low level of financial knowledge compared to male counterparts mainly due to the principal role of FDM being shouldered by the male members of the family. Interestingly, Bernasek and Bajtelsmit [34] concluded that the income earned by women has a direct impact on FDM, implying a nexus between the degree of FDM involvement and the income earned. Arguments have also arisen on how women engage at par with men in daily money management affairs while refraining from major FDM which entail specialized financial knowledge, acumen, and capability [30,32]. With respect to financial attitude (FA), Cornwall et al. [35] opine that female are inclined albeit successful in decisions offering immediate rewards in comparison to male counterparts who fare better in delayed rewards tasks. In addition, females prefer consistent rewards while males focus on the magnitude of rewards.

More recently, Hsu et al. [36], conclude the power of financial literacy to equalize behavioural bias in financial decision making across both genders. Nevertheless, the take on IMP and FAUT is limited and inconclusive with available findings voicing female dissuasion from impulsivity as they demonstrate diligence in saving money for family and specifically the educational advancement of their children [37]. On the contrary, women perceive FAUT as a cushion to guard during impending contingencies [38] mainly in situations where they outlive their partners. Extending our support to the former arguments, we assume that gender acts as a moderator in strengthening/weakening the impact of FDM determinants on FDM. Hence, we propose the following hypothesis.

H1 Males and Females significantly differ in their financial decision making (FDM)

### 2.2 Digital financial literacy (DFL)

The proliferation of digital financial services and platforms have aggravated the relevance of digital financial literacy (DFL) over financial literacy. Time has proven that financial literacy alone cannot help one to wade through the complexities in the digital world. Unfortunately, DFL is now an imperative for individuals to access financial products and services or make financial decisions. It refers to the knowledge, skill, and abilities to access digital financial services [39]. The former authors also stress on various dimensions of DFL as the awareness of digital financial services, ability to use digital platforms, ability to make digital financial transactions and to make rational decisions without succumbing to online fraudulent practices. Although research is silent on the underpinning theory behind this concept, we argue that ‘gain goal frame’ under the goal framing theory [40] can provide a fair support to the DFL concept where the criterion for realizing goals (i.e., sound financial decisions) necessitates the enhancement of resources (DFL being the intangible resource here).

The goal framing theory posits that individuals endeavour to advance their goals which is over all wellbeing by engaging in various self-regulating behaviour. Furthermore, the theory assumes that human beings try to achieve conflicting/multiple goals, thereby self-motivating them to be engrossed in higher order cognitive functions. These multiple goals are then grouped as goal frames and decision-making behaviour is then ruled by any one or all the goal frames namely hedonic, gain and normative. While the hedonic goal frame focuses on the present feel good notion, the gain goal frame is related to judicious decision making which assures conserving resources and increasing income. The normative goal frame on the other hand refer to those heuristic behaviour that emerge from external factors. However, goal frames are not mutually exclusive, and the theory postulates the strength of a goal frame being confronted by the individual's current goals arising from unexpected situations [41]. For example, a financially literate individual who has a saving mentality (gain goal frame) might indulge in a money splurge or unwise investments due to impulsivity (hedonic goal frame).

Therefore, it should be noted that the goal frames extent of assertion in decision making is a cumulative effect of external factors as well the capacity to regulate one's behaviour. Here we stress that DFL will equip individuals with the confidence and skills to use financial platforms and services effortlessly, thereby removing the mental barriers that inhibit the usage and access towards such services. It will also enable users to intensify their digital financial management usage over simple internet usage [42] and take wise financial decisions such as abstinence from overspending albeit enhancing savings [14] and minimising excessive borrowing. Sound DFL will also dissuade individuals from being prone to online risks and fraudulent practices such as phishing, pharming, catfishing, and others. Enabling the availability of essentials at one's fingertips, DFL will be immensely useful to attain financial capability and independence while growing in age and preparing to minimise their dependency on children. However, DFL being a nascent concept, very few papers [43–45] have surfaced in this domain and none till date on the direct DFL-FDM nexus. Hence, we suggest the following hypothesis.

H2 Digital financial literacy (DFL) positively relates to financial decision making (FDM)

### 2.3 Financial capability (FC)

Financial capability (FC) is a multi-dimensional concept that encompasses both judicious money management and rational decision making where individuals carefully weigh the expected costs with the benefits whilst being mindful of their financial constraints [46]. However, multiple perceptions have evolved regarding the precedents of FC. While Atkinson et al. [47] assign financial literacy as the precedent, another school of thought considers behavioural attributes to play a vital role in impacting FC and thereby financial decisions [48]. In continuum, Bruggen et al. [27] opine that a blend of individual's experience and educational background with FC leads to varied financial behaviours while financial inclusion and community characteristics [49], positive FA [50] and knowledge of financial markets [51] emerge as other significant precursors of FC, henceforth affecting FDM.

The concept has been underpinned by the capability approach, pioneered by Amartya Sen and Martha Nussbaum in 1980's who proposed FC as an outcome of the external socio-cultural environment one is exposed to apart from being the conversion factor which transforms available resources (e.g., money) into valuable resources (pension) [52]. Prior research has minimally investigated FC, either in terms of its determinants [53] or as a mediator in financial well-being [54]. Thus, we argue that FC equips one with the ability and opportunity to take prudent financial decisions based on their accumulated knowledge about the financial process, financial products and services that provides them wide accessibility to a range of financial services. We concur to Atkinson et al. [47] that financial literacy or more specifically DFL acts as a vital precursor to FC and arms individuals with the confidence and motivation to manage financial activities, thereby enhancing their FC. The latter then leads to individual empowerment furnishing them with financial cognitive skills, resilience, and confidence to sagaciously associate with financial service providers by taking sound financial decisions for converting dormant resources into invaluable resources with wise financial decisions. Therefore, the hypotheses are formulated as follows.

H3 Financial capability (FC) positively relates to financial decision making (FDM)

H4 Financial capability (FC) significantly mediates the relationship between digital financial literacy (DFL) and financial decision making (FDM)

### 2.4 Financial attitude (FA)

The current paper explores financial attitude (FA) as a psychological factor impacting financial decision in three forms, namely decision to consume, decision to save or decision to invest [13]. FA is a measure of the mental state, judgement, and opinion towards finance, having an element of agreement or disagreement [55] whilst contributing to one's financial success or failure in decision making [56,57]. It is also perceived as the application of financial principles to make value-based decisions and optimize resource management [58]. It traces its roots to the theory of reasoned action [59] which propounds that financial behaviour (which is FDM in this study) is the outcome of an intention (financial wellbeing), the former emanating from one's attitude towards the same.

Furnham [60] outlines six dimensions or reasons for variations in FA which are obsession towards money, money as a power mechanism, enhancing self-worth, fear of fund inadequacy, need for liquid cash and savings mentality. Prior research has attested the positive significant impact of FA on FDM [13]. We add on to the argument that FA entails perceptional differences towards finance as elaborated in Furnham's [60] dimensions. Individuals with positive FA add financial insight, craft informed financial decisions in the form of controlled expenses, retirement plans and judicious investments due to their appropriate usage of financial principles and inherent quest for acquiring financial knowledge and literacy. Thus, we propose that.

H5 Financial attitude (FA) has a positive relation with financial decision making (FDM)

### 2.5 Impulsivity (IMP)

Impulsivity, according to Barberis and Thaler [61], is a psychological trait which warrants further investigation to unwrap the cognitive psychology that aids in FDM, apart from the conventional determinants such as financial literacy. Whiteside and Lynam [62] identified perseverance (lack of), urgency, sensation seeking and premeditation (lack of) as the four main drivers of IMP. Premeditation which falls under the functional class [63] is the inclination to foresee the consequences of the choice made prior to making choices. However, lack of premeditation (falling under the dysfunctional class) leads to faulty and irrational FDM due to minimal degree of control, foresight and deliberation exercised by the individual [64]. Later, Zermatten et al. [65] also attested the impact of premeditation on IMP.

Dickman and Meyer [63] argued that two-dimensional theory paints a positive and negative picture of IMP. Costa and McCrae [66] emerged with a five-factor model (extraversion, neuroticism, agreeableness, openness to experience, and conscientiousness) as the apt theory to rationalize IMP and its relationship with FDM, in the wake of the futility of other theories (as listed in Ref. [67]) in modelling a comprehensive theory to underpin IMP. Till date, IMP has been studied as a mediator between financial literacy and debt decision making [15,68] as an outcome of financial education [69] or as a moderator between financial literacy and FDM [54]. However, to the best of researchers’ awareness, there is no record of any study that delved into the direct impact of IMP on FDM or its moderating effect in the FC-FDM nexus.

As an attempt to bridge the aforesaid gap, we argue that IMP (i.e., a psychological outcome of dysfunctional dimension, non-agreeableness, extraversion, and unconscious efforts) drives an individual to unplanned, hasty, and suboptimal financial decisions to reap short term benefits without looking at the potential negative long-term impact of the decision. IMP leads to risky financial decisions such as over-spending and over indebtedness [70] as emotions have overpowered rationality either to obtain immediate sense gratification, alignment with the herd behaviour or to engage in self-indulgence. Interestingly, IMP among low-income groups have recurrently proved to be higher in relation to spending on junk food [71]. Also, we assert that IMP can downplay the positive effects of FC on FDM and hence weaken the relationship.

Early studies have already factored in this variable [72] in behavioural finance and exerted that financial distress and dissatisfaction (on faulty FDM) are evident in the presence of IMP regardless of the financial education and literacy possessed. This is because IMP robs individuals' perceived ability to control their actions (locus of control) as well as future planning propensities [73], negating the positive attributes embedded in FC and paving way for suboptimal FDM [54]. We assert that the power of IMP overpowers one's FC, succumbing to irrational, hasty and short-term oriented decisions. We therefore formulate the following hypotheses.

H6 Impulsivity (IMP) negatively relates to financial decision making (FDM)

H7 Impulsivity (IMP) significantly moderates the relationship of financial capability (FC) with financial decision making (FDM)

### 2.6 Financial autonomy (FAUT)

Financial autonomy (FAUT) refers to the limited dependency on others albeit having the capability and liberty to engage in FDM. As a research topic, the nexus of FAUT as a driver of FDM has remained under-explored [74]. Vosylis and Klimstra [16] perceive FAUT as a quality which develops in stages and regard it as the degree of responsibility one shoulders for decisions related to managing daily spending, purchasing and investment choices. The concept has been backed by the goal framing theory [40] which purports that realization of one's goals (i.e., FDM here) inevitably calls for the enrichment on one's resources (i.e., FAUT here). Adding on, we assume that individuals amass this trait either through socialization [16] or self-motivation and consciously enhances it while encountering several FDM decisions throughout their life.

Jariwala and Dziegielewski [75] add that this motivation empowers people to adopt strategic changes in their decisions as well as evaluate savings – investment choices subsequently leading to positive and fruitful financial decisions. This is because, humans are rational beings who like to be independent decision makers with minimal errors and maximum returns. On another note, Luo et al. [12], used the term financial independence instead of FAUT to reflect the relevance of economic, educational as well as psychological factors in impacting FAUT, and we are in concurrence with this viewpoint.

That said, the research in FAUT-FDM nexus is limited, as extent research has either unwrapped the determinants of FAUT [16] or explored the impact of financial socialization on financial FAUT [74]. This paper will fill this void and emerge as the pioneer in exploring the direct impact of FAUT on FDM on the premise that the FAUT enables independent and rationale FDM due to the experience amassed from family interactions, awareness of technological advancements, constant upskilling, self-empowerment, enhanced decision-making competency and the evolving confidence and responsibility to take ownership of one's actions without being dependent on others. Therefor the following hypothesis is formulated.

H8 Financial autonomy (FAUT) positively relates to financial decision making (FDM)

## 3 Methodology

### 3.1 Data collection and sample

To investigate the determinants of FDM, we conducted a questionnaire-based survey. The empirical data was collected from the respondents located in the National Capital Territory (NCT) of Delhi, India using the mix of random and snowball sampling. Since this study requires a respondent to be an investor, thus a mandatory check question was added, i.e., whether the respondent invests in any kind of financial instrument and accordingly respondents were allowed to proceed further only when the answer was ‘yes’. The Likert 5-point measurement scales were used to test the theory, in alignment with the previous research (see Annexure 1). The questionnaire was managed using google forms which elaborated the objective of the study in the introduction section. The responses were collected between February 2022 and August 2022.

The survey clearly mentioned about the voluntary participation of the respondents (due consent was taken from the respondents before proceeding with the survey) and assured that their responses would be treated with utmost confidentially whilst maintaining anonymity. The willingness to complete the questionnaire is an expression of respondent's consent to take part in the research. It was compulsory to attempt all questions that were asked in the google form, eliminating any chances of having missing data. The questionnaire consisted of three sections i.e., independent variables (DFL, IMP, FA, and FAUT) in section one; mediator (FC) and dependent variable (FDM) in section two; demographic profile in section three. The study focused on Delhi as it was the worst Covid hit area with the exponential rise in cases leaving the city far behind the financial capital Mumbai which was once the largest COVID hotspot in the country. This led to further lockdowns, thus crippling the economy, and leading to catastrophic repercussions on financial decision making and wellbeing.

The sample size sufficiency was ensured by the G*Power software via a priori analysis [76]. With a statistical power of 0.80, effect size 0.15 and seven predictors, the minimal sample indicated by G*power was 103 and as suggested by Hair et al. [77], one should take 3 times this suggested sample size. The final sample after cleaning data for the unengaged participants and outliers, was 634, thus meeting the minimum sample size requirement estimated by the G*Power [77,78].

### 3.2 Measures and scales

Financial autonomy (FAUT) was conceptualized as the higher order construct comprising of three lower order reflective constructs or dimensions namely emotional, functional, and reflexive. The questionnaire and scales used in the present study has been adopted from previous studies on the topic of research, as presented in Table 1.

**Table 1:** Questionnaire scales.

Scale	Source(s)
Digital financial literacy (DFL)	Morgan and Trinh [44] and Muellabuer [79]
Financial attitude (FA)	Shockey [80]
Financial autonomy (FAUT)	Micarello et al. [81]
Financial capability (FC)	Atkinson et al. (2007)
Impulsivity (IMP)	Shockey [80]
Financial decision making (FDM)	Lizarraga et al. [82]

The scales have been measured on five-point Likert scale, 1 representing strongly agree and 5 strongly disagree. Annexure 1 shows the items with respect to each of the construct. All these scales have high validity and reliability, as shown in Table 3.

### 3.3 Demographics

Table 2 highlights that out of the total 634 respondents received during the survey period, 54% of the respondents were male and 46% were female. Of the sample respondents, 25% worked in private companies, 31% were academicians and 15% were students pursuing undergraduate and postgraduate courses. 83% of the respondents were in the age group of 21–50 years. With private and public employee, we meant respondents working in any private and public sector undertaking respectively, excluding the educational institutions. The respondents who are in the profession of teaching be it in any private or public educational institution were included in academician category only.

**Table 2:** Demographic profile.

Characteristics	Classification	Frequency	Percent
Gender			
	Male	342	54%
	Female	292	46%
Profile			
	Student	95	15%
	Academician	197	31%
	Businessman/woman	114	18%
	Private employee	159	25%
	Public employee	69	11%
Age			
	<20 years	63	10%
	21–30 years	222	35%
	31–40 years	159	25%
	41–50 years	146	23%
	51–60 years	38	6%
	>60 years	6	1%

### 3.4 Data analysis

To assess the common method bias, the bivariate correlations between the constructs has been checked and all values were found to be less than 0.90 [83]. As a second check, the variance inflationary factor (VIF) values have been estimated and found to be less than 3.3 [84], implying absence of common method bias (Table 3).

The SmartPLS 3.3 [85] has been used to perform the hypothesis testing and PLS predict was applied to estimate the out sample predictive power. The PLS-SEM approach has a broad scope and is flexible regarding theory and practice [86]. It is used to evaluate theoretical and complicated models and assess the predictive ability of the proposed model. For conducting the multi-group analysis (MGA), measurement invariance has been assessed using the MICOM procedure [87]. Henseler's MGA and the permutation test has been then used to estimate the multi-group analysis (MGA) results [88].

## 4 Results

SmartPLS 3.3 software [85] has been used to run the PLS algorithm, to assess the reliability and validity of the construct, with the default settings of path as the weighing scheme and maximum iterations = 300. Bootstrapping technique was then run to test the hypothesis with 10,000 subsamples, complete bootstrapping and the corresponding 95% bias-correlated and accelerated confidence interval [89]. The disjoint two stage approach has been adopted in this study because of the presence of reflective-reflective higher order constructs (HOC) [86]. In the first stage, the PLS algorithm has been run with all the lower order constructs (LOC) without the presence of HOC. Then the latent variable scores for emotional, reflexive, and functional were saved. In the second stage, HOC has been formed with emotional, reflexive, and functional LOC scores as its indicators. The remaining constructs has been measured item-wise and used as the LOC only.

### 4.1 Reliability and validity

As presented in Table 4, all the composite reliability (CR) and rho_A values were greater than 0.70, hence confirming the reliability of the indicator. The average variance extracted values (AVE) were also found to be greater than the threshold of 0.50, indicating the presence of convergent validity [86]. The factor loadings of almost all the indicators were greater than the threshold of 0.708 [90]. However, some lower factor loadings were retained due to good reliability and validity of the construct.

The constructs exhibited satisfactory discriminant validity (see Table 5) with all Heterotrait-Monotrait ratio (HTMT) of correlation being either <0.85 or <0.90 [86,91]. The Disjoint two stage approach resulted in beta values corresponding to path coefficients between the LOC and the HOC (financial autonomy). The results shown in Table 3 exhibit presence of good reliability and satisfactory validity for the financial autonomy. All the paths showed the positive and significant loadings at 1% level of significance (see Fig. 2).

**Table 3:** Multicollinearity assessment using VIF.

Constructs	VIF value
Digital Financial Literacy	1.336
Financial Attitude	2.077
Emotional	2.618
Functional	2.946
Reflexive	2.458
Financial Capability	1.780
Impulsivity	1.060

### 4.2 Multi-group analysis

As per the existing literature studies, FDM is influenced by gender. Thus, to analyse this, we have conducted the multi-group analysis (MGA) based on the gender of a respondent. To conduct MGA, the measurement invariance must be established using the MICOM procedure [87,88]. The configural invariance was established, as in both the groups (male and female) the items used to measure a construct were same. After that, with parametric tests at 1000 permutations, compositional invariance was also established. Thus, conducting MGA for further analysis is considered meaningful as full measurement invariance was established (see Table 6).

**Table 4:** Measurement model results.

Construct & Items	Loadings	Cronbach's α	rho_A	CR	AVE
Digital Financial Literacy (DFL)		0.924	0.936	0.936	0.619
DFA1	0.773				
DFE1	0.779				
DFE2	0.755				
DFE3	0.769				
DFK1	0.738				
DFK2	0.848				
DFK3	0.813				
DFK4	0.839				
DFK5	0.760				
Financial Attitude (FA)		0.930	0.938	0.942	0.647
FA1	0.842				
FA2	0.850				
FA3	0.842				
FA4	0.765				
FA5	0.845				
FA6	0.621				
FA7	0.767				
FA8	0.868				
FA9	0.808				
Emotional (EMOT)		0.766	0.782	0.847	0.581
FA_E1	0.796				
FA_E2	0.739				
FA_E3	0.789				
FA_E4	0.724				
Functional (FUNC)		0.863	0.870	0.901	0.646
FA_F1	0.794				
FA_F2	0.780				
FA_F3	0.805				
FA_F4	0.795				
FA_F5	0.844				
Reflexive (REFL)		0.833	0.835	0.900	0.751
FA_R1	0.899				
FA_R2	0.889				
FA_R3	0.808				
Financial Capability (FC)		0.943	0.943	0.954	0.746
FC1	0.854				
FC2	0.876				
FC3	0.833				
FC4	0.900				
FC6	0.900				
FC7	0.855				
FC8	0.826				
Financial Decision Making (FDM)		0.933	0.936	0.946	0.715
FDM1	0.793				
FDM2	0.841				
FDM3	0.886				
FDM5	0.857				
FDM6	0.879				
FDM8	0.798				
Impulsivity (IMP)		0.844	0.855	0.905	0.761
IM1	0.842				
IM2	0.899				
IM3	0.874				
Financial Autonomy (FAUT)		0.876	0.878	0.924	0.802
EMOT	0.891*				
FUNC	0.915*				
REFL	0.880*				

Note: * Indicates significant at 1% level of significance.

**Table 5:** Discriminant validity.

	DFL	FA	EMOT	FUNC	REFL	FC	FDM
FA	0.366						
EMOT	0.429	0.701					
FUNC	0.383	0.692	0.873				
REFL	0.416	0.677	0.801	0.864			
FC	0.442	0.597	0.636	0.589	0.595		
FDM	0.547	0.593	0.649	0.585	0.575	0.860	
IMP	0.217	0.184	0.184	0.124	0.078	0.097	0.208

**Table 6:** Result of MICOM (step 2 & 3).

Full measurement invariance established		Yes	Yes	Yes	Yes	Yes	Yes	Yes
Equal variance assessment	Equal	Yes	Yes	Yes	Yes	Yes	Yes	Yes
	CI	[-0.336; 0.340]	[-0.622; 0.671]	[-0.452; 0.467]	[-0.377; 0.396]	[-0.374; 0.387]	[-0.236; 0.227]	[-0.619; 0.637]
	Differences	−0.032	0.216	0.047	0.091	0.286	0.159	0.106
Equal mean assessment	Equal	Yes	Yes	Yes	Yes	Yes	Yes	Yes
	CI	[-0.216; 0.220]	[-0.216; 0.226]	[-0.218; 0.225]	[-0.216; 0.223]	[-0.219; 0.223]	[-0.222; 0.220]	[-0.257; 0.262]
	Differences	0.182	0.071	0.074	−0.047	0.213	−0.001	0.058
Partial measurement invariance established		Yes	Yes	Yes	Yes	Yes	Yes	Yes
Compositional variance		0.996	0.998	1	0.999	1	0.993	1
Configural invariance		Yes	Yes	Yes	Yes	Yes	Yes	Yes
Construct		DFL	FA	FAUT	FC	FDM	IMP	FC*IMP

MGA was meaningful for our study with 342 (54%) male and 292 (46%) female. The standard guidelines related to group sample size differences suggest that if a certain group is 50% larger than the other, the difference is likely to bias the results of the statistical test of differences [92].

The p values in the last column of Table 7 indicate significance levels of differences between path coefficients of males and females. Taking the MGA into consideration, the difference in p values indicated insignificant difference for all the structural paths. It implies that there was no significant difference between male and female FDM. Hence, the H1 was not supported by the results. The direct relation of DFL with FDM is more in case of males as compared to females (β = 0.185* → 0.180*). The same is the scenario in case of direct relation of FAUT (β = 0.126* → 0.167^ns^) and FC (β = 0.657* → 0.590*) which is strong and significant in case of males. Although FAUT relation becomes insignificant in case of females (β = 0.167^ns^), IMP has been negatively related to FDM in both the groups, with more significance in case of females (β = −0.155* → −0.106*). However, moderating effect of IMP on the FC and FDM relationship becomes insignificant across both the groups, with similar results being applicable to FA as well. Thus, the full sample results have been considered for the study.

**Table 7:** Results of MGA based on 5000 permutations.

Hypothesis/Structural path	Path Coefficient		Path Coefficient Difference	p-value difference	Result
	Male	Female			
DFL → FDM	0.185*	0.180*	0.005	0.956	Rejected
FA → FDM	0.076^ns^	−0.024^ns^	0.100	0.407	Rejected
FAUT → FDM	0.126*	0.167^ns^	−0.041	0.347	Rejected
FC → FDM	0.657*	0.590*	0.067	0.615	Rejected
IMP → FDM	−0.106*	−0.155*	0.049	0.261	Rejected
FC*IMP → FDM	−0.047^ns^	−0.087^ns^	0.040	0.592	Rejected
DFL → FC → FDM	0.307*	0.234*	0.073	0.460	Rejected
R^2^	0.686	0.611			

Note: ‘*’ indicates significant at 5% level of significance; ‘ns’ indicates not significant.

### 4.3 Structural model assessment

The bootstrapping technique was conducted to evaluate the hypothesis with 10,000 subsamples, the corresponding 95% bias-corrected confidence interval [89]. In the pooled sample (Table 8), all the determinants except FA (H5) were found to be significant at 5% level of significance. DFL, FAUT, FC, and IMP were positively associated with the FDM, thus failing to reject H2, H3, and H6. The total effect of DFL on FDM was 0.454 and indirect effect was 0.259, thus VAF (variance accounted for) was 0.57 (0.259/0.454). H4 was also supported by the results, i.e., FC positively and significantly mediates the relationship between DFL and FDM (β = 0.259; [0.183–0.344]).

**Table 8:** Structural model assessment of full sample.

Path	β (Full)	T stat	BC CI (L) 5%	BC CI (U) 95%	Result
H2: DFL → FDM	0.195*	4.075	0.116	0.273	Fail to Reject
H3: FC → FDM	0.607*	9.314	0.503	0.716	Fail to Reject
H4: DFL → FC → FDM	0.259*	5.265	0.183	0.344	Fail to Reject
H5: FA → FDM	0.029ns	0.501	−0.065	0.125	Reject
H6: IMP → FDM	−0.102*	2.706	−0.132	−0.040	Fail to Reject
H7: FC*IMP → FDM	−0.070*	2.047	−0.127	−0.017	Fail to Reject
H8: FAUT → FDM	0.116*	2.010	0.012	0.212	Fail to Reject
	${\text{R}}_{\text{full}}^{2}$ = 0.690				

Note: ‘*’ indicates significant at 5% level of significance; ‘ns’ indicates not significant; ‘BC CI (L)’ – Bias corrected confidence interval (lower); ‘BC CI (U)’ – Bias corrected confidence interval (upper); DFL – digital financial literacy; FDM – financial decision making; FC – financial capability; FA – financial attitude; IMP – impulsivity; FAUT – financial autonomy.

**Fig. 3:**
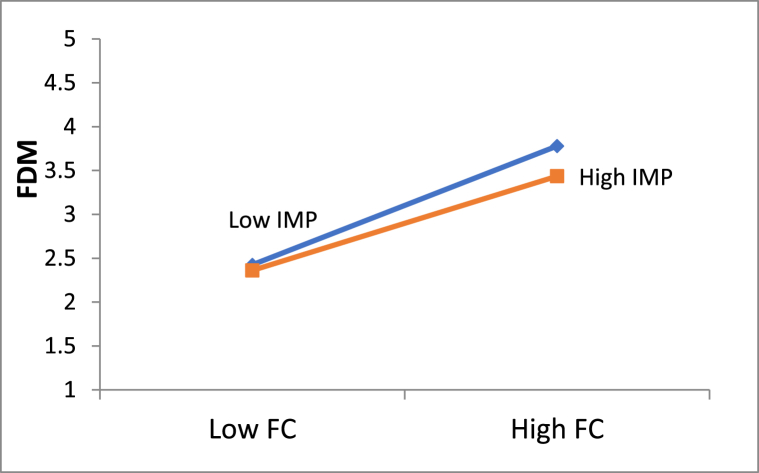
Moderating effect of IMP. *Source: Authors' own estimates*.

Fig. 3 represents the simple slope analysis of the moderating effect of IMP on the relationship between FC and FDM (β = −0.070*). The IMP dampens the positive relationship between FC and FDM. In other words, relationship between FC and FDM was found to be negatively moderated by IMP. Hence, we fail to reject H7, i.e., IMP negatively moderates the relationship of FC and FDM.

After assessing the overall structural model by way of bootstrapping, the researchers have also assessed the predictive ability of the proposed model. The out sample predictive power has been estimated using the PLS Predict with 10 folds and 10 repetitions. Table 9 shows that the errors estimated by the PLS were less as compared to the linear model (LM – naïve benchmark). Since for all indicators of FDM, the difference between PLS and LM RMSE (i.e., errors) were negative, thus model has high out-sample predictive power.

**Table 9:** PLS Predict results.

	PLS_RMSE	LM_RMSE	Difference	Q^2^_predict
FDM1	1.382	1.462	−0.08	0.166
FDM2	1.187	1.244	−0.057	0.276
FDM3	1.269	1.336	−0.067	0.299
FDM5	1.341	1.487	−0.146	0.274
FDM6	1.272	1.362	−0.09	0.288
FDM8	1.372	1.409	−0.037	0.243

## 5 Discussion

The current study contemplates behavioural, psychological, and demographic factors impacting financial decision making (FDM) as an outcome of the interaction among individual behavioural differences, life experiences, practices, and formal instruction with each one developing content knowledge, attitude, competencies and skills towards financial topics and financial domain. The first hypothesis proposed varied trends in FDM in males and females, but results report a contradiction to the former, thus signalling gender irrelevance in FDM [93]. This can be associated with rising trends noticed in engagement parity, as evident in financial management affairs, by empowering women with resources to enhance their decision-making power thus scaling down the male dominance in FDM and upscaling the dormant powers vested in women, all of which narrow down the gender gap in rational FDM. That said, the evident dominating independent positive effects of DFL and FAUT in male FDM cannot be ignored and clearly evince the relevance of the effect, both the former factors hold in enhancing FDM. As Fonseca et al. [32] emphasised, the willingness to harness DFL leads to specialized financial knowledge and capability, both of which confer them financial independence to take sound financial decisions. Here we argue that males inevitably shoulder this responsibility of acquiring financial knowledge/literacy to cater to family requirements with women silently acknowledging their efforts and diverting one's concentration in non–financial matters [30].

In continuum, hypothesis 2 assumes a direct relationship in the DFM-FDM nexus with results emerging statistically significant to attest the former proposition. We express our congruence to the goal framing theory and findings of Azeez and Akhtar [43], where the authors emphasise the positive influence of DFL in various financial behaviours paving way for effective FDM. This reiterates the myriad benefits DFL confers in the form of effortless financial management, using digital platforms, improved confidence in identifying fraudulent practices, resilience building and enhanced FC to independently handle financial matters; all of which will reassure robust FDM in the form of profitable investments, boosted savings and mindful borrowing.

The next set of hypotheses (H3 and H4) investigated the direct and mediating relation of FC with FDM. The results confirm to the hypothesized positive relationship apart from reflecting a resemblance to the findings of Tahir et al. [54]. To substantiate H3 and H4, we assert that FC endows confidence, independence, and motivation to engage in FDM independently and wisely. This also indicates the classic role of the capability approach which accentuates FC building in conjunction with digital skills, all which function as forerunners to sound financial administration leading to optimal financial decisions. The supplementing efforts of DFL cannot be side-lined as it fortifies existing financial capacities whilst equipping them with knowledge to access various digital financial platforms that will assist one in enhancing FDM [45].

Embarking on other determinants of FDM, H5 proposes a positive association between FA and FDM with results contradicting the same with the emergence of an insignificant relationship in the FA-FDM connection. This can be attributed to the respondents’ profile which majorly consist of young individuals who are yet to develop perceptional differences towards FDM in terms of retirement decisions and long-term investments. The findings also contradict the revelations of Arifin et al. [13] amongst others, all of whom reporting a positive FA-FDM nexus.

The effects of IMP on FDM as well as the moderating effects of IMP in the FC-FDM relationship were the next focal points of attention in H6 and H7, with results conforming the hypothesized negative relationship between IMP and FDM. This is because, IMP diminishes adequate forethought and erodes logical buying behaviour, replacing it with irrational short-term self-gratification, all of which pave way for substandard FDM [73]. Therefore, the authors restate the relevance of integrating psychological factors to unveil the ingrained cognitive psychology which unconsciously guides human financial behaviour. Moving on, results for IMP as a moderator in the FC-FDM connection clearly reveal the deleterious effects of impulsivity in overpowering the accrued benefits emanating from FC, thus leading to irrational FDM. We therefore suggest that despite being financially capable due to acquired financial literacy, IMP can overshadow and undue the benefits from the former. This is because, impulsive behaviour leads to disorganized, unstructured, and illogical hasty decisions which can be the outcome of several factors mentioned in Costa and Mc Craes's [66] five factor model, just to quell short term demands which seldom are worthwhile in future.

Lastly, H8 presumes a positive relationship between FAUT and FDM, the results of which have emerged statistically significant to accept the alternative hypothesis. Aligning our results to goal framing theory as well as the findings of Jariwala [74] who advocate a vital role played by financial autonomy in influencing FDM, we reason that individuals today are well informed and knowledgeful about the cumulative benefits of informed decision making, thus taking initiatives to harness financial and experiential skills to mitigate financial constraints faced. During this journey of gaining FAUT, they want to exhibit their authenticity of skills gained, take independent and wise financial decisions after weighing the pros and cons, marginal benefits, and cost, all to ensure recognition in society as sound financial decision makers. The overall results of this comprehensive and unique study portray the relevance various psychological behavioural and demographic factors bear on FDM, all of which favour the design of a feasible and lucrative financial portfolio to ensure financial well-being in the long run [75].

## 6 Conclusion

This study reiterates the importance of adopting an integrative approach for better decision making where each system can derive optimal results under varying situations, hence corroborates the recommendation provided by Hochman et al. [20]. This is done by: (a) exploring the roles of the psychological and behavioural factors as unconscious but significant contributors to FDM, (b) investigating the nexus between these factors and FDM and extending the radius of the existing literature, (c) evaluating the moderating effects of gender on the determinants of FDM, and (d) finally drawing implications from the empirical results.

In our literature review, we came across various schools of thought and formulated eight hypotheses for testing the nexus of FDM with a list of psychological and behavioural factors, such as positive FAUT, FA including IMP and materialistic attitude, sound DFL and FC as cumulative effects of experience, skills, and attitude, as well as mediating roles of selective factors in the nexus. Moreover, as a major area of focus, we investigated the gender influence on FDM, and observed limited and inconclusive take of researchers on IMP and FA and hence examined varied trends in FDM from the gender perspectives but found no significant FDM behavioural difference between males and females.

Based on our empirical findings, we can claim making some contributions to the extant literature on FDM. For example, we observed a positive association of DFL, FAUT, and IMP with FDM. Further, we observed a significant positive mediating role of FC in the DFL-FDM nexus. Based on our literature review, we however failed to trace any research that has explored any FC-FDM nexus or a mediating influence of FC in the DFL-FDM nexus. Given that DFL is a nascent concept, hence very few papers have surfaced in this domain and none till date has attempted to make any empirical investigation on the direct nexus of DFL with FDM. We claim the incorporation of DFL in this study and evidence of its influence on FDM, are a contribution to the existing literature. Moreover, this study investigated the direct impact of IMP (e.g., financial distress and dissatisfaction) on FDM and its moderating effect in the FC-FDM nexus.

We argued that IMP overpowers one's FC and drives an individual to unplanned, hasty, and suboptimal financial decisions to reap short term benefits, without looking at the potential negative long-term impact of the decision (FDM). We spotted a negative moderation effect of IMP in the FC-FDM relationship and hence claim another contribution to the extant literature. Further, we investigated varied trends in FDM in males and females but found no significant difference between them in FDM. Given that the direct effect of DFL on FDM is more in case of males as compared to females, we emphasise creating more DFL opportunities for women. Moreover, as a pioneering study to decipher a new empirical model to comprehend the FDM process, we claim to fill some voids in the extant literature given that prior studies [94,95] only examined the individual influence of the FDM factors and missed to check their total impact on FDM.

### 6.1 Limitations, recommendation for future research

Although the sample and the research design adopted in this study are adequate to achieve the desired research objectives, like any other research, this study is also susceptible to few limitations. The sample size sufficiency was ensured by the G*Power software in this study. However, given that provinces/states in India are highly diverse in nature, we believe that use of a larger size of sample with respondents from various states could provide a more robust picture of the FDM behaviour of an average citizen in India and enable greater generalisability of the findings in the contexts of the developing world with similar socio-economic features. Also, given that “countries with higher rates of savings have had a faster economic growth than those with lower saving rates” [96], further research may include institutional respondents like NGOs, cooperatives, community investment schemes, and so on to develop insights of how a sound financial management of household savings can be made and channelled towards contributing to the overall economic growth of a populous country like India. The inherent limitations of cross-sectional data, especially the inadequate evidence of temporal association between exposure and outcome is also assumed to appear in this study.

This study provides an empirical and conceptual springboard for ensuing work on other potentially important determinants of FDM behaviour. Given that behavioural finance is a relatively new but a fast growing field where the list of psychological factors will continue to expand and new opportunities and challenges arise, further studies can be planned by considering other psychological factors such as regret bias, gender IMP, anchoring effect, defence mechanisms, personality traits (e.g., self-esteem, self-identity, etc.), over and under reaction, mental accounting, and so on to examine their likely impacts on investors’ FDM attitudes. As successful research outcomes, many of these factors might turn out to be useful determinants of the risk-taking and FDM behaviour of the household investors.

Also, in the wake of ongoing automations and digital transformations of financial services, we emphasise further studies on exploring relevant behavioural factors such as prejudice and general mindset to DFL, emotional intelligence, among others and their influence on FDM, with particular focus on gender bias. Also, in consideration of the fact that the global digital skills crisis is worsening due to growing automation and use of artificial intelligence [97], and digital financial services and platforms are fast proliferating, we emphasise prioritising DFL over financial and numeric literacy and disseminating the knowledge through popular digital means, e.g., social media platforms. This approach will not only equip individuals with the confidence and skills to use financial platforms and services effortlessly, but also dissuade individuals from being prone to online risks and fraudulent practices and refrain them from impulsive (IMP) behaviour.

In order to develop further insights into this topic and minimise the rising knowledge gap in literature with regard to the nexus between gender and FDM [98], it would be useful to conduct empirical research on the gender-specific FDM-behaviour including IMP and check various socio-economic scenarios such as: (a) the influence of different decision-making styles (e.g., male- or female-dominant, syncretic/joint, autonomous) including level of participations in dyadic dynamics between males and females as partners or married couples; (b) the current state of financial knowledge and/or literacy and capability of partners in optimal FDM in the wake of fast technological progress and high speed of change; (c) the perception and influence of individual or shared financial goals and wellbeing of partners on decision making on savings, insurance, buy-in, & others.

### 6.2 Implications

The findings in this research would enable financial regulators and policymakers to better understand the nature and type of the influence that psychological and behavioural factors have on household investors’ FDM. In the ongoing post-pandemic recovery period, this research will be beneficial for financial professionals, regulatory bodies, or investment advisors in understanding the behavioural patterns in FDM that are likely to be associated with the economic and financial market volatilities. This study also will help investors to be aware of the impact of their own psychological factors on their FDM and accordingly enhance the rationality and efficiency of their investment decisions. Also, given that we have attempted to minimise the differential gap between India and similar developing economies from a demographic perspective by investigating the gender-FDM nexus in the context of India, we emphasise that further exploration about this relationship in connection with the listed behavioural and psychological factors will enable household investors to understand their market behaviour better and make candid decisions regarding their investments from inheritances and/or family savings.

## Author contribution statement

Rekha Pillai: Analyzed and interpreted the data; Wrote the paper.

Parul Kumar: Conceived and designed the experiments; Performed the experiments; Analyzed and interpreted the data; Wrote the paper.

Md Aminul Islam: Contributed reagents, materials, analysis tools or data.

Taimur Sharif: Contributed reagents, materials, analysis tools or data; Wrote the paper.

## Funding statement

This research did not receive any specific grant from funding agencies in the public, commercial, or not-for-profit sectors.

## Data availability statement

Data will be made available on request.

## Declaration of interest's statement

The authors declare no conflict of interest.
