# Supplementary material for: Analysing the behavioural, psychological, and demographic determinants of financial decision making of household investors
Source: Heliyon. 2023 Jan 21;9(2):e13085. doi: 10.1016/j.heliyon.2023.e13085 (PMC9922930; doi:10.1016/j.heliyon.2023.e13085)
Supplement: Multimedia component 1 [file mmc1.docx]

**Annexure 1**

| Construct / Items | |
| --- | --- |
| **DIGITAL FINANCIAL LITERACY(DFL)** | |
| **Digital Financial Awareness** | |
| **DFA1** | Awareness about the potential of financial risk in using digital financial provider or fintech, such as the legality of the fintech provider, interest rate and transaction fee |
| **Digital Financial experience** | |
| **DFE1** | Having experience in using the product and service of fintech for digital payment |
| **DFE2** | Experience in using the product and service of fintech for financing (loan) and investment |
| **DFE3** | Experience in using the product and service of fintech for asset management |
| **Digital Financial Knowledge** | |
| **DFK1** | Having a good understanding of digital payment products such as E-Debit, E-Credit, E-Money, Mobile/Internet banking, E -wallet |
| **DFK2** | Having a good understanding of product digital asset management |
| **DFK3** | Having a good understanding of digital alternatives |
| **DFK4** | Having a good understanding of digital insurance |
| **DFK5** | Having a good understanding of customer rights and protection as well as the procedure to complain about the service from digital financial providers |
| **FINANCIAL CAPABILITY (FC)** | |
| **FC1** | I am very organized when it comes to managing my money daily |
| **FC2** | I keep a close personal watch on my financial affairs |
| **FC3** | I always make sure I have money saved up for emergency/unexpected expense |
| **FC4** | I do a good job of balancing my spending and saving |
| **FC6** | I am good at dealing with day-to-day financial matters |
| **FC7** | I feel confident about the financial decision I make |
| **FC8** | I make certain I understand the commitments I agree to in financial contracts |
| **IMPULSIVITY** | |
| **IM1** | I often do things without giving them much thought |
| **IM2** | I am impulsive |
| **IM3** | I say things before i have thought them through |
| **FINANCIAL AUTONOMY(FAUT)** | |
| **Emotional** | |
| **FA_E1** | I take part in domestic expense planning |
| **FA_E2** | I usually have a critical view of the way my friends deal with money. |
| **FA_E3** | I like to participate in family decision making when we buy something expensive for home. |
| **FA_E4** | I advice others on money matters |
| **Functional** | |
| **FA_F1** | I always try to save some money to do things I really like |
| **FA_F2** | I always like to negotiate prices when I buy |
| **FA_F3** | I suggest at home that we keep money aside for emergencies |
| **FA_F4** | I keep an eye on promotions and discounts |
| **Reflexive** | |
| **FA_R1** | I like to think thoroughly before deciding to buy something |
| **FA_R2** | I like to research prices whenever I buy something |
| **FA_R3** | I pay attention to news about the economy as it may affect my family. |
| **FINANCIAL ATTIUDE (FA)** | |
| **FA1** | It is important for me to control monthly expenses. |
| **FA2** | It is important for me to establish financial targets for the future |
| **FA3** | It is important for me to save money on a monthly basis. |
| **FA4** | The way I manage my money today will affect my future. |
| **FA5** | It is important for me to have and follow a monthly expense plan. |
| **FA6** | It is important for me to pay the full value on credit cards. |
| **FA7** | When buying in installments, it is important to compare available credit offers. |
| **FA8** | It is important for me to stay within a budget. |
| **FA9** | It is important for me to invest regularly to achieve targets in the long term. |
| **FINANCIAL DECISION MAKING (FDM)** | |
| **FDM1** | I am able to quickly change my financial decisions as per the changes in circumstance. |
| **FDM2** | Appraise of personal risk helps me in better financial decision making |
| **FDM3** | I make sound financial decision by comparing results over the time |
| **FDM5** | I make sound financial decisions by comparing results over expenses involved. |
| **FDM6** | I am able to search for economic options during financial decision making |
| **FDM7** | I am able to foresee the long term and short term consequences of the financial decisions I undertake |
| **FDM8** | Previously used decision strategies helps me in better financial decision making |
